# Influence of Light Wavelength and Optotype Size on Accommodative Response and Aberrometric Changes Across the Adult Lifespan

**DOI:** 10.1007/s44402-026-00036-0

**Published:** 2026-03-09

**Authors:** Inés Cabrera-Guardiola, Elvira Orduna-Hospital, María Arcas-Carbonell, Ana Sanchez-Cano

**Affiliations:** 1 Department of Applied Physics, University of Zaragoza, Zaragoza, Spain; 2 Aragon Institute for Health Research (IIS Aragon), Zaragoza, Spain

**Keywords:** Age-related accommodation response, Chromatic stimuli, Pupil diameter, Target size, Wavefront aberrometry, Zernike coefficients

## Abstract

**Purpose:**

To analyse the effects of chromatic light (white, blue, red and green) and stimulus size (6/6 and 6/12) on pupil constriction, Zernike coefficients and the accommodative response curve using wavefront aberrometry across a wide age range of healthy subjects.

**Methods:**

One hundred and sixty-four right eyes from participants aged 20–75 years were evaluated. All subjects showed normal near visual function for their age. Wavefront aberrations were measured under scotopic conditions using the IRX3 aberrometer. Accommodation was induced from 0 to 10 D in the younger group and from 0 to 5 D in the full sample. Stimuli varied in colour and size. Pupil diameter and Zernike coefficients were analysed, rescaling all maps to a 3.00 and a 3.65 mm pupil, respectively.

**Results:**

Mean pupil diameter ± standard deviation decreased progressively with increasing accommodative demand by 0.51 ± 0.06 mm in the full sample (0–5 D) and by 2.09 ± 0.11 mm in the younger group (0–10 D). The greatest changes were observed under white light and larger stimuli. The Zernike component *C*(2,0) varied significantly across all filters, optotype sizes and in both the total (*p* < 0.003) and the younger (*p* < 0.0009) groups. However, *C*(4,0) showed significant changes in all conditions for the younger group (*p* < 0.0009), particularly at higher demands. An initial overaccommodation of approximately 1 D at baseline was followed by a progressive lag beyond 5 D, being more pronounced under red light, where the accommodative response was lowest, while white light consistently elicited the strongest response. Larger stimuli induced greater responses than smaller ones, especially at high demands.

**Conclusion:**

Accommodation efficiency varies with wavelength and stimulus size: white and blue lights triggered greater pupil constriction and accommodation than red and green, with corresponding changes in defocus and spherical aberration. Small stimuli improved low-demand responses, while larger ones were more effective at higher demands.

Key Points

- White and blue light produced greater pupil constriction and more accurate focusing than red and green light during visual tasks.
- The size of the visual stimuli affected the eye’s ability to focus, depending on whether the focusing demand was low or high.
- Higher-order aberrations changed with colour and target size, while focusing accuracy decreased at higher stimulus demands and under red or green illumination.

## Introduction

Accommodation is the physiological process by which the crystalline lens changes shape to adjust ocular power and focus at different distances, mediated by ciliary muscle contraction [1, 2]. The lens’s focusing ability can be affected by longitudinal chromatic aberration (LCA), as different wavelengths of light focus at different points (from blue to red), causing up to 2 dioptres (D) of defocus under broadband white light [3]. While LCA can reduce image quality in polychromatic light [4, 5], as well as contrast sensitivity [6–8], it also increases the depth of field, potentially influencing the accommodation process [5, 9]. This enhanced depth of field has been proposed as a possible explanation for the nonlinearity observed in accommodation, suggesting that different wavelengths may be focused depending on object distance [10]. However, this hypothesis has been refuted by subsequent studies [11, 12]. LCA may also provide a directional cue for accommodation, helping the visual system detect whether to increase or decrease focus based on colour blur differences [13]. Some studies [4, 14] have shown that reducing chromatic aberration or using monochromatic light impairs dynamic accommodation accuracy but does not affect static accommodation significantly. This suggests that LCA is more important for initiating focus changes than for maintaining focus [11, 15]. However, most previous studies have evaluated accommodative responses at fixed viewing distances [4, 14, 16], and only a few have examined the full range of responses under narrow-wavelength monochromatic conditions [17].

Ocular wavefront aberrations arise when the ideal wavefront is distorted, causing image blur on the retina [18]. These aberrations are quantified using Zernike polynomials [19], a set of orthogonal functions that describe optical errors by radial order and azimuthal frequency [20]. Low-order aberrations (LOAs) (*n* = 0, 1 and 2) are linked to basic refractive errors, while high-order aberrations (HOAs) (*n* ≥ 3), such as coma, trefoil and spherical aberration [20], contribute to retinal image degradation [21].

Wavefront changes during accommodation have been widely studied [1, 22–24], as significant structural modifications occur in the crystalline lens during focus adjustment [2]. With ageing, these alterations become more pronounced, reducing accommodative amplitude and contributing to presbyopia [25]. Additionally, finite-element modelling confirms that age-related stiffening of lens components is correlated with diminished accommodative amplitude [26, 27]. Although these changes have been widely studied in young adults, the accommodative response and its optical consequences can vary substantially across the ageing visual system. Over time, lens stiffening and loss of transparency affect both the effectiveness of accommodation and the generation of optical aberrations, underscoring the importance of examining these phenomena across different age groups, which is essential for interpreting visual dynamics properly under physiological conditions and in the context of early presbyopia [28, 29].

Wavefront changes induced by accommodation are not limited to the Zernike defocus coefficient [30], but involve multiple coefficients that vary in magnitude. Among these, spherical aberration shows the most significant changes, typically shifting from positive to negative values during accommodation [22, 31–34]. In contrast, other higher-order coefficients, such as vertical and horizontal coma, tend to show reduced variability, with no clear systematic changes [32, 35] or alternatively demonstrate relative stability across different accommodative conditions [24, 31, 36].

Previous work has shown that both chromatic content and spatial properties of the visual stimulus modulate accommodative accuracy [22, 30]. While LCA provides directional cues for focus [30], the detectability of the target is strongly influenced by its angular size and spatial frequency [32]. However, the interaction between chromatic cues and target size has not been examined systematically, despite their potential to jointly influence the precision and dynamics of accommodation. Therefore, combining wavelength and optotype size within the same experimental design allows the determination of whether these two independent cues interact, enhancing or limiting accommodative efficiency responses across ages.

The present study aimed to determine whether chromaticity and optotype size influence accommodative accuracy independently or through interactive mechanisms. To address this, their effects on pupil constriction, wavefront aberrations and accommodative responses were examined across a broad adult age range, using stimuli from 0 to 10 D in younger participants and 0 to 5 D in the full sample. We hypothesise that short-wavelength (blue) and broadband (white) stimuli will elicit greater accommodative accuracy and stronger pupillary constriction than longer-wavelength stimuli (red or green), since short-wavelength light focuses in front of the retina and provides stronger directional cues for the visual system to detect and correct defocus through LCA. Additionally, we predict that optotype size modulates accommodative dynamics, with smaller targets (6/6) promoting higher precision at low accommodative demands and larger targets (6/12) supporting sustained responses at higher demands. Finally, we expect these chromatic and spatial effects to diminish with age, reflecting the physiological decline in accommodation and chromatic sensitivity associated with presbyopia.

## Methods

### Sample Description and Selection

The study adhered to the principles of the Declaration of Helsinki and was approved by the Clinical Research Ethics Committee of Aragón (CEICA) under reference number PI23-439. All participants received comprehensive information about the study protocol and provided written informed consent prior to enrolment.

A total of 164 healthy participants aged between 20 and 75 years were included in the study, comprising 86 women (52.44%) and 78 men (47.56%). Eligibility criteria required participants to have best-corrected visual acuity (VA) of 6/6 (0.00 logMAR) or better in the right eye, measured with an Early Treatment of Diabetic Retinopathy Study chart at 4 m for distance and at 40 cm for near vision. The amplitude of accommodation (AA) was assessed monocularly using Donders’ push-up method. Additionally, monocular accommodative facility with a ± 2 D flipper at 40 cm, the near point of convergence and vergence facility (VF) with a 3Δ base-in /12Δ base-out flipper prism at 40 cm were evaluated. Participants presenting with any accommodative dysfunction (including accommodative insufficiency, excess or infacility) were excluded.

Refractive error limits were defined as follows: spherical errors outside the range of ±6.00 D and astigmatism >1.50 D were exclusionary. Axial length (AL) was required to be between 22 and 27 mm. Prior to each session, participants were instructed to abstain from wearing contact lenses and to avoid stimulants (caffeine, alcohol or tobacco) and electronic device use for at least 2 h.

Only data from the right eye of each participant were included in the analysis to prevent redundancy due to binocular symmetry. Participants from the total group (TG), 20–75 years of age (*n* = 164) were stratified into five age-based subgroups for analysis: Group 1 (G1), 20–29 years (*n* = 82); Group 2 (G2), 30–39 years (*n* = 20); Group 3 (G3), 40–49 years (*n* = 20); Group 4 (G4), 50–59 years (*n* = 22) and Group 5 (G5), 60 years and older (*n* = 20). This age-based grouping was implemented to ensure an even distribution of participants across the non-young age range (G2–G5: 30–75 years) and to confirm that all subjects met normal visual function criteria for their respective age group. In the analysis, this five-group classification was used specifically for the analysis of accommodative response curves, to account for the known decline in accommodation with age. However, the main results for Zernike coefficients are presented for the young (G1) and total (TG) groups, which better reflect the variations in Zernike coefficients across the full age spectrum.

### Ocular Parameter Assessment and Experimental Procedure

AL was measured using the IOL Master 500 (Carl Zeiss Meditec, zeiss.com/meditec), averaging five consecutive readings per subject. Ocular aberrations were assessed under controlled scotopic ambient lighting conditions (0 lux) using the IRX3 aberrometer (Imagine Eyes, imagine-eyes.com/), which employs Hartmann–Shack wavefront sensing with a 780 nm infrared beam and a 32 × 32 microlens array. The system includes a Badal mechanism to modify the stimulus vergence over a 35 D range while maintaining a constant angular size of the target.

**Fig. 1:**
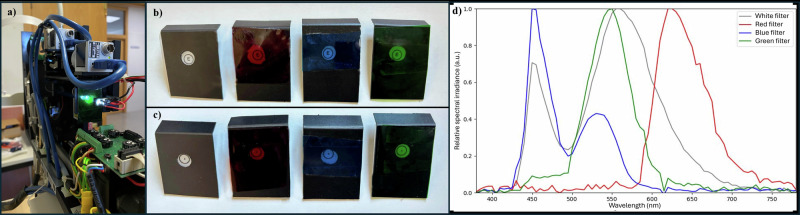
Experimental setup, stimulus holders and spectral characteristics of the chromatic filters. **a** Experimental setup of the adapted IRX3 aberrometer (Imagine Eyes, imagine-eyes.com/), featuring one of the custom-designed holders used to position the visual stimuli and optical filter during measurements. **b**, **c** Holders used for the visual stimuli with optotypes corresponding to Snellen E 6/12 and 6/6 sizes, respectively. Each holder incorporates a white, red, blue or green filter positioned adjacent to the optotype. **d** Emission spectra of the optical filters measured at the corneal plane, with wavelength (nm) represented on the *x*-axis and relative spectral irradiance (a.u. arbitrary units) on the *y*-axis.

Measurements were conducted monocularly on the right eye, with the fellow eye fully occluded. Participants fixated on a black Snellen E optotype (6/6 or 6/12), presented against a coloured background (white, red, green or blue) through custom-designed filter holders (Fig. 1). The optotype was imaged through a Badal optical system, which optically projected the target to each participant’s far point. All targets were calibrated for size at the testing distance, ensuring identical measurement conditions across all filters and stimulus sizes. Each participant completed a single session lasting approximately 20–25 min, with short breaks introduced between filter conditions to minimise fatigue and accommodative decay. To avoid order and learning effects, the sequence of chromatic filters and optotype sizes was randomised across participants, and both optotype sizes were completed for each filter before advancing to the next condition, ensuring controlled intra-subject comparisons.

Spectral power distribution and total irradiance (W m^−2^) of the filters were measured using a calibrated spectroradiometer (StellarNet Black-Comet, StellarNet Inc., stellarnet.us/). Luminance at the image plane was assessed with a luminance metre (Mavo-Spot 2, gossen-photo.de), resulting in values of 250 cd/m² (white), 50 cd/m² (blue), 40 cd/m² (green) and 5 cd/m² (red). Although luminance was not equalised, this approach ensured faithful spectral separation without introducing additional filters.

The study was divided into two parts based on the participants’ age groups. For G1, composed of young adults aged 20–29 years, aberrometric measurements were obtained for accommodative stimuli ranging from 0 to 10 D in 1 D increments, resulting in 11 steps for each experimental condition (stimulus colour and size). Prior to the test, participants were instructed to fixate on the central and distinguishable part of the stimulus to enhance fixation stability. A 2-s interval was provided between each step to allow sufficient time for accommodative adjustment, leading to a total recording time of 22 s. In contrast, older participants (G2–G5, ranging from 30 to 75 years of age) underwent aberrometric recordings using a dynamic accommodative stimulation protocol with six vergence steps from 0 to 5 D in 1 D increments, also with a 2-s fixation period per step. In all groups, participants were allowed to blink freely during measurements to minimise optical artefacts related to tear film instability [37, 38]. In participants over 60 years of age, accommodative amplitude was naturally minimal (<1 D), yet full stimulus-response curves were recorded to characterise age-related dynamics and ensure methodological consistency across groups.

During the aberrometric measurements, no refractive correction was used. Instead, the spherical equivalent (*M*) of each participant’s refractive error was determined in a baseline measurement to establish the initial accommodative state of each eye. To ensure relaxation of accommodation, the IRX3 aberrometer employed a masking technique [24, 32] presenting the stimulus at 1 D beyond the participant’s far point (determined through refraction derived from Zernike coefficients), while continuously monitoring the ocular refractive state. Based on this *M* value, the aberrometer automatically computed the wavefront aberrations for the 11 (G1) or 6 (G2–G5) levels of accommodative demand. Ocular aberrations were initially determined at the pupil plane and exported for the largest circular pupil size, expressed as Zernike coefficients *C*(*n, m*).

Given that accommodation affects the pupil diameter [39–42], a standardised pupil size was used to enable accurate comparison across different measurements. This diameter was defined as the value for which 95% of eyes exhibited a larger pupil at that accommodative state (5th percentile). For G1, where accommodation ranged from 0 to 10 D, a standardised pupil diameter of 3.00 mm was applied based on this criterion. When accommodation was only stimulated up to 5 D, a standardised diameter of 3.65 mm was selected following the same procedure. These values were determined from the data presented in Tables 2 and 3. The wavefront data corresponding to eyes above the 5th percentile in each case were rescaled to the selected pupil diameters using the method described by Schwiegerling [43] and refined by Visser et al. [44]. Multiple text files (.txt) were generated and stored in a hierarchical folder structure organised by subject, filter, stimulus size and accommodative level. These files were subsequently imported into Excel databases (Microsoft® Office Excel 365, microsoft.com/en-us/microsoft-365/excel).

### Statistical Analysis

Data processing, statistical analysis and visualisation were carried out using a custom Python programme (python.org) developed in Google Colab (colab.research.google.com), which analysed the Excel-based datasets. A descriptive analysis (mean, standard deviation, maximum and minimum) was performed for key Zernike coefficients sensitive to accommodation. As normality (assessed via the Kolmogorov–Smirnov test) was not met, non-parametric tests were applied. The Friedman test was used to assess differences across the four filters, followed by post-hoc, Bonferroni-corrected comparisons. A significance threshold of *p* < 0.0009 was used when comparing accommodative steps from 0 to 10 D (0.05/55 possible pairwise combinations) and *p* < 0.003 when comparing steps from 0 to 5 D (0.05/15 combinations).

Spearman correlation coefficients (*ρ*) were calculated to explore associations between Zernike coefficients across accommodative states. Linear regressions were also performed to examine their relationship with the non-accommodative baseline. A significance level of *p* < 0.05 was set.

Additionally, the actual accommodation achieved was estimated from Zernike coefficients and compared with the induced demand for each filter and optotype size. To assess differences between the four filters (white, blue, red and green) at each level of accommodative demand, the Friedman test was applied, followed by post-hoc, Bonferroni-corrected comparisons, using a significance threshold of *p* < 0.008 (0.05/6 possible filter pairings).

### Ethical Approval

The study was conducted in accordance with the Declaration of Helsinki and approved by the Clinical Research Ethics Committee of Aragón (CEICA), reference PI23-439.

## Results

A total of 164 healthy right eyes were evaluated, with ages ranging from 20 to 75 years and a mean age of 36.25 ± 15.78 years. The mean AA in G1 was 11.04 ± 2.61 D (Table 1). Accommodative and vergence function tests were administered to all participants across the age groups. As expected, older participants exhibited limitations in accommodative facility due to the age-related decline in accommodation, as reflected in Table 1.

**Table 1:** Descriptive parameters of the sample regarding the number of subjects (*n*), gender with the number of males and females (*M*/*F*), mean ± standard deviation axial length (AL), anterior chamber depth (ACD), spherical equivalent (*M*), amplitude of accommodation (AA) and monocular accommodative facility (MAF) from the right eye and binocular vergence facility (VF), considering the total group (TG) and by age groups (Group 1 (G1), 20–29 years (*n* = 82); Group 2 (G2), 30–39 years (*n* = 20); Group 3 (G3), 40–49 years (*n* = 20); Group 4 (G4), 50–59 years (*n* = 22) and Group 5 (G5), 60 years and older (*n* = 20)).

	*n*	Age (years)	Gender (*M*/*F*)	AL (mm)	ACD (mm)	*M* (D)	AA (D)	MAF (cpm)	VF (cpm)
TG	164	36.25 ± 15.78	78/86	24.47 ± 1.32	3.35 ± 0.37	−1.69 ± 1.32	7.01 ± 5.60	7.72 ± 7.35	10.83 ± 4.82
G1	82	22.93 ± 2.73	36/46	24.02 ± 1.25	3.64 ± 0.32	−1.58 ± 1.82	11.04 ± 2.61	13.33 ± 4.24	11.22 ± 5.17
G2	20	35.67 ± 2.28	10/10	24.48 ± 1.08	3.53 ± 0.28	−2.56 ± 1.97	9.31 ± 5.86	7.10 ± 7.78	10.93 ± 4.71
G3	20	45.50 ± 3.60	10/10	25.31 ± 0.83	3.49 ± 0.38	−3.45 ± 2.79	1.63 ± 2.59	1.56 ± 4.22	11.31 ± 4.82
G4	22	54.64 ± 2.81	12/10	24.3 ± 1.73	3.25 ± 0.32	−0.97 ± 2.48	0.73 ± 0.99	0 ± 0	10.57 ± 5.03
G5	20	63.42 ± 4.43	12/8	23.56 ± 0.58	2.97 ± 0.26	−1.95 ± 2.41	0.47 ± 1.00	0 ± 0	10.23 ± 4.48

### Pupil Diameter

Analysis of pupil size revealed consistent variations influenced by accommodative demand, stimulus size and spectral composition of the light (Tables 2 and 3). In both experimental groups, dynamic stimulation from 0 to 10 D in G1 only and from 0 to 5 D in groups G1–G5, the pupil diameter decreased progressively as accommodative demand increased. However, the extent and pattern of this constriction differed depending on the colour filter and optotype size. In the G1 group (0–10 D), the red filter produced the largest pupil diameters for both 6/12 and 6/6 stimuli, whereas the white filters induced the most pronounced pupillary constriction. Blue and green filters elicited intermediate responses, with the green filter generally resulting in slightly larger pupil diameters than the blue. These trends were consistent across all levels of accommodative demand. In G1–G5, assessed from 0 to 5 D, similar patterns were observed. The red filter consistently yielded the largest pupil diameters, followed by green, blue and white. Importantly, the smaller optotype (6/6) led to greater pupillary constriction than the larger one (6/12), likely due to increased retinal luminance. This effect suggests a significant role of stimulus size in modulating the pupil response, alongside the spectral properties of the stimulus.

**Table 2:** Mean pupil diameter ± standard deviation (SD) in millimetres and 5th percentile values for each level of accommodation (from 0 to 10 D) and optotype evaluated (Snellen E 6/12 and Snellen E 6/6 size in white, blue, red and green) for Group 1 with 82 subjects.

Group 1 (*n *= 82)		Snellen E 6/12											Snellen E 6/6										
		0 D	1 D	2 D	3 D	4 D	5 D	6 D	7 D	8 D	9 D	10 D	0 D	1 D	2 D	3 D	4 D	5 D	6 D	7 D	8 D	9 D	10 D
WHITE	Mean	6.61	6.47	6.29	6.13	6.07	5.85	5.57	5.29	5.03	4.76	4.60	6.47	6.39	6.32	6.18	6.06	5.84	5.59	5.29	4.93	4.74	4.50
	±SD	1.02	1.05	1.09	1.14	1.15	1.15	1.17	1.11	1.05	0.90	0.82	1.10	1.08	1.10	1.13	1.14	1.15	1.10	1.08	0.94	0.87	0.77
	5th percentile	4.64	4.51	4.19	4.21	4.16	3.90	3.58	3.42	3.21	3.11	3.05	4.41	4.54	4.45	4.19	3.97	3.90	3.71	3.46	3.41	3.29	3.03*
BLUE	Mean	6.72	6.61	6.52	6.46	6.30	6.10	5.81	5.44	5.15	4.86	4.72	6.69	6.58	6.54	6.47	6.33	6.11	5.78	5.50	5.16	4.85	4.54
	±SD	1.00	1.03	1.10	1.11	1.13	1.12	1.08	1.09	0.93	0.84	0.75	1.01	1.10	1.08	1.10	1.16	1.11	1.16	1.05	0.94	0.83	0.77
	5th percentile	5.00	4.76	4.40	4.73	4.43	4.36	4.15	3.58	3.72	3.31	3.26	4.82	4.57	4.77	4.67	4.26	4.10	4.02	3.70	3.56	3.40	3.12
RED	Mean	6.99	6.82	6.78	6.67	6.62	6.31	6.09	5.82	5.47	5.17	4.86	7.01	6.93	6.84	6.69	6.52	6.36	6.04	5.77	5.36	5.01	4.72
	±SD	0.96	1.10	1.00	1.16	1.10	1.20	1.09	0.98	0.94	0.89	0.78	0.98	1.03	1.06	1.15	1.12	1.15	1.12	1.04	0.87	0.81	0.75
	5th percentile	4.77	4.51	4.95	4.41	4.30	3.75	3.88	3.72	3.66	3.72	3.59	5.33	4.66	4.66	4.53	4.28	3.91	3.95	3.87	3.88	3.52	3.20
GREEN	Mean	6.78	6.64	6.56	6.46	6.35	6.10	5.83	5.50	5.13	4.90	4.65	6.73	6.61	6.51	6.39	6.26	6.02	5.83	5.50	5.22	4.92	4.72
	±SD	0.95	0.98	1.04	1.04	1.03	1.00	1.02	0.96	0.85	0.75	0.71	1.02	1.05	1.09	1.12	1.11	1.15	1.14	1.06	0.98	0.84	0.76
	5th percentile	5.12	4.69	4.78	4.64	4.59	4.36	4.21	3.97	3.69	3.57	3.40	4.74	4.63	4.35	4.46	4.22	4.18	4.10	3.80	3.63	3.62	3.50

The 5th percentile smallest value was used as a reference to standardise pupil size in the analysis (marked in bold with*).

**Table 3:** Mean pupil diameter ± standard deviation (SD) in millimetres and 5th percentile values for each level of accommodation (from 0 to 5 D) and optotype evaluated (Snellen E 6/12 and Snellen E 6/6 size in white, blue, red and green) for the total group with 164 subjects.

Total group (*n* = 164)		Snellen E 6/12						Snellen E 6/6					
		0 D	1 D	2 D	3 D	4 D	5 D	0 D	1 D	2 D	3 D	4 D	5 D
WHITE	Mean	6.08	5.91	5.81	5.70	5.64	5.46	5.92	5.84	5.80	5.67	5.62	5.45
	±SD	1.14	1.18	1.15	1.14	1.17	1.15	1.18	1.17	1.18	1.19	1.15	1.16
	5th percentile	4.32	3.92	4.03	3.90	3.80	3.76	4.18	3.96	3.98	3.85	3.82	3.71
BLUE	Mean	6.16	6.07	6.00	5.93	5.84	5.71	6.16	6.07	6.02	5.96	5.85	5.67
	±SD	1.15	1.14	1.17	1.18	1.16	1.13	1.17	1.20	1.21	1.20	1.22	1.16
	5th percentile	4.15	4.06	4.03	3.97	3.98	3.78	4.10	4.14	3.90	3.97	3.84	3.67
RED	Mean	6.38	6.26	6.24	6.16	6.10	5.91	6.39	6.32	6.28	6.15	5.99	5.86
	±SD	1.14	1.18	1.14	1.20	1.19	1.19	1.16	1.17	1.19	1.25	1.26	1.22
	5th percentile	4.3	4.22	4.32	4.18	3.95	3.75	4.34	4.41	4.46	3.85	3.63	3.72
GREEN	Mean	6.21	6.08	6.00	5.95	5.85	5.71	6.17	6.03	5.95	5.88	5.78	5.62
	±SD	1.13	1.14	1.17	1.14	1.14	1.07	1.15	1.21	1.22	1.21	1.20	1.18
	5th percentile	4.28	4.10	3.98	4.04	3.90	3.75	4.27	4.03	3.91	3.95	3.73	3.65*

The 5th percentile smallest value was used as a reference to standardise pupil size in the analysis (marked in bold with*).

Based on the values presented in Table 2, the smallest pupil diameters in G1 (*n* = 82) were observed at the 5th percentile with a 10 D accommodative demand using the white filter: 3.05 mm for the 6/12 stimulus and 3.03 mm for the 6/6 optotype. Consequently, a reference pupil diameter of 3.00 mm was established for rescaling all aberrometric data in this first phase. In contrast, the smallest pupil diameter in G1–G5 (*n* = 164) occurred with the green filter under a 5 D accommodative demand using the 6/6 stimulus (5th percentile: 3.65 mm), Table 3. Therefore, a standardised pupil diameter of 3.65 mm was used for wavefront rescaling in this second phase. This approach ensured the comparability of aberrometric measurements across all filters, stimulus sizes and age groups.

### Distribution of Zernike Coefficients Measured With the Aberrometer

**Fig. 2:**
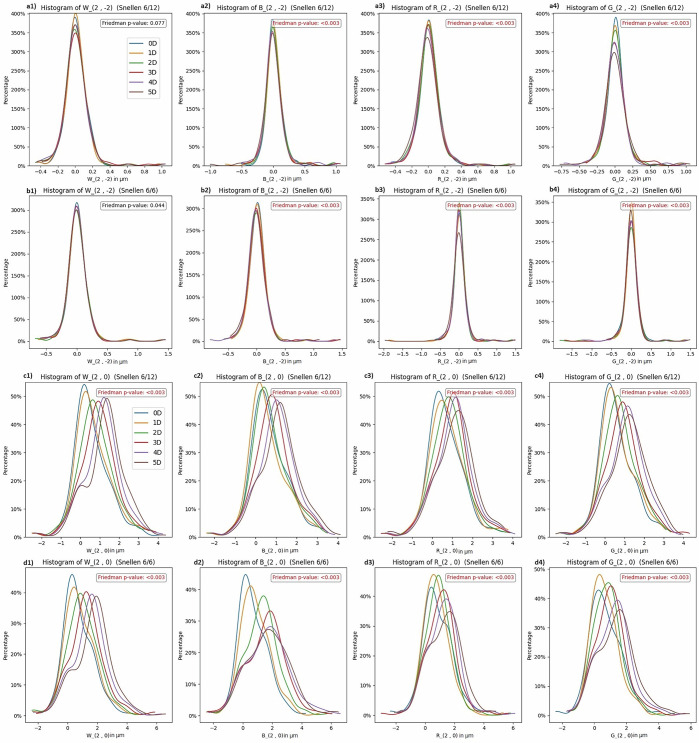

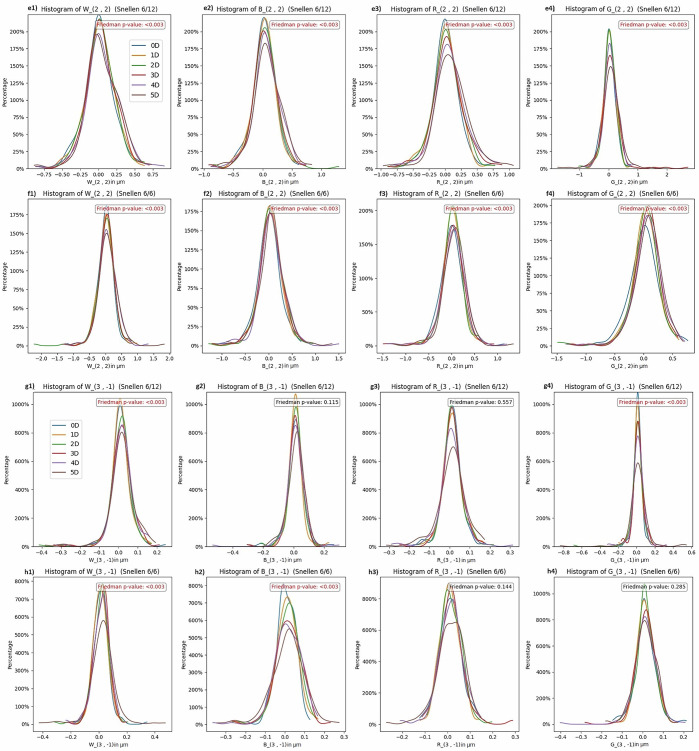

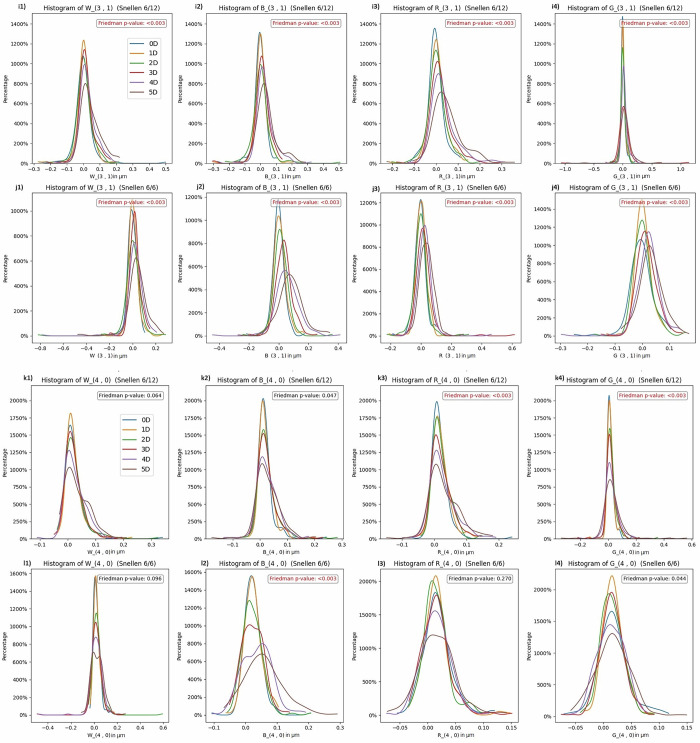

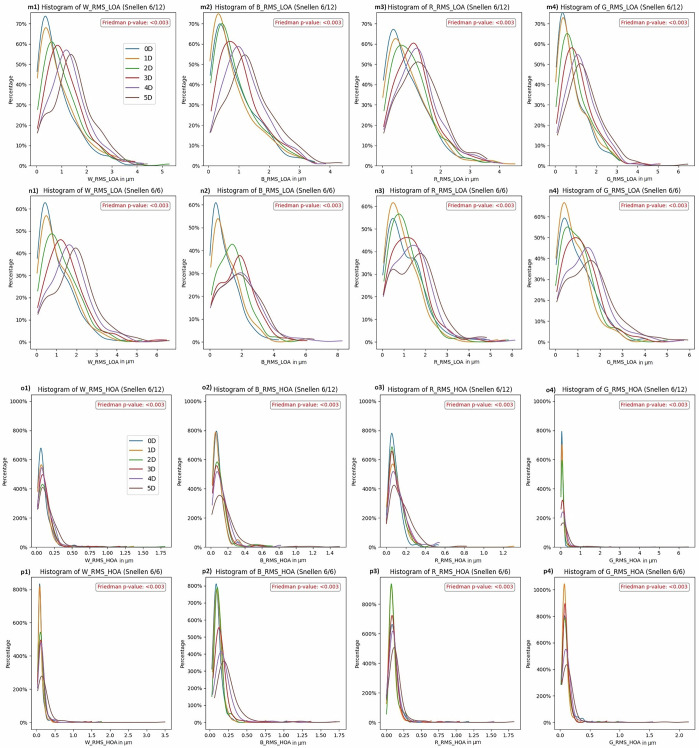
(Continued)

**Fig. 3:**
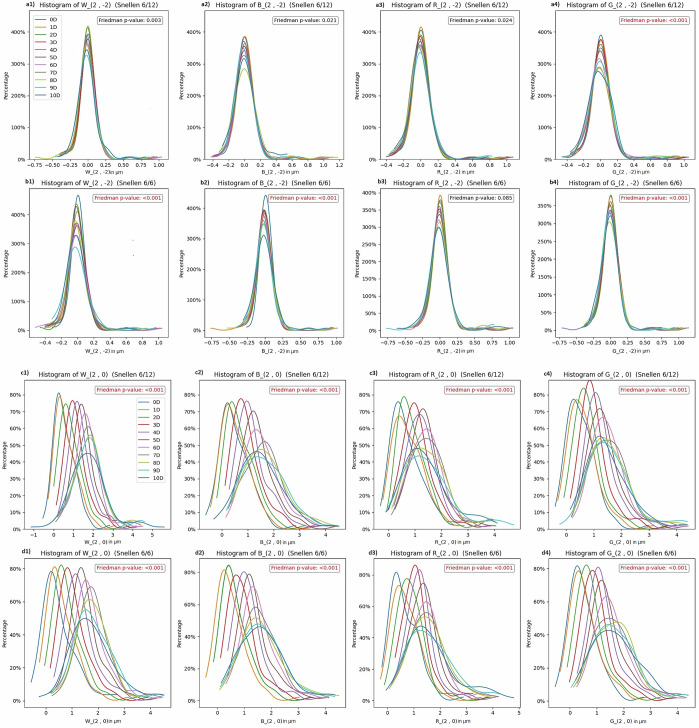

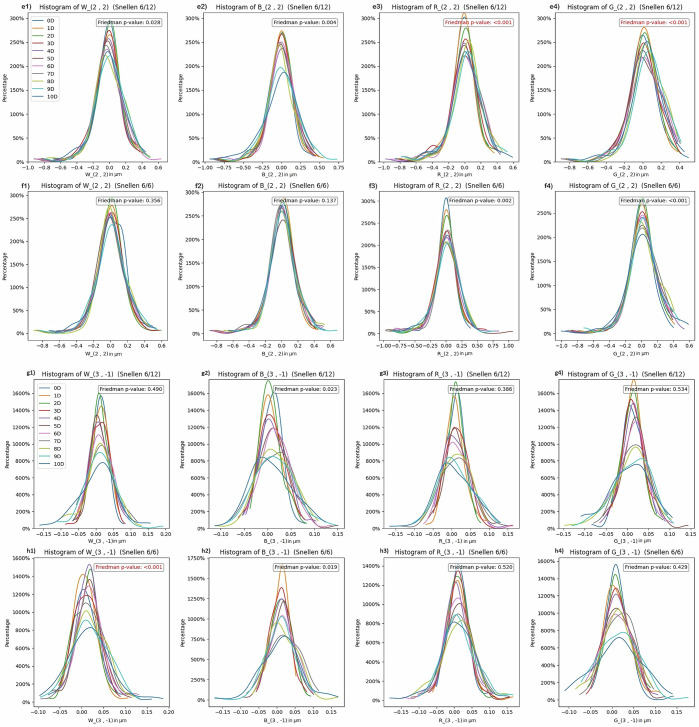

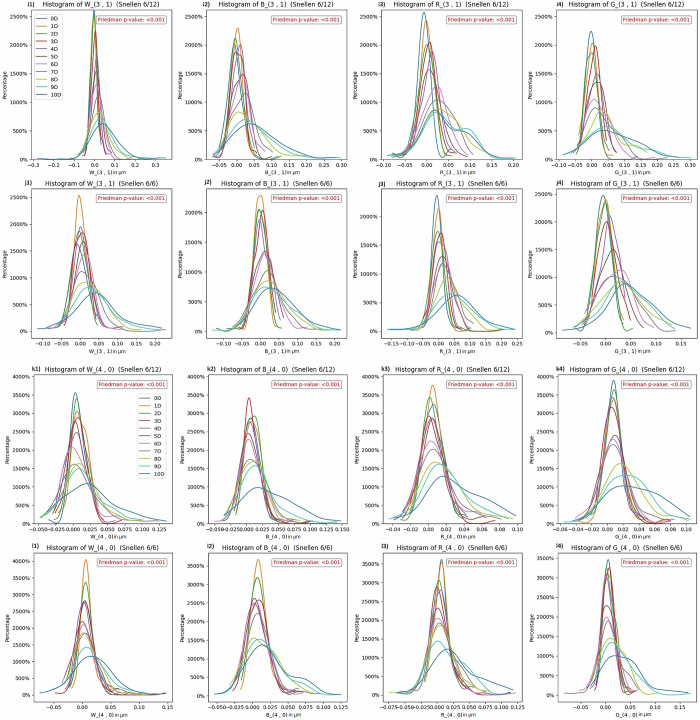

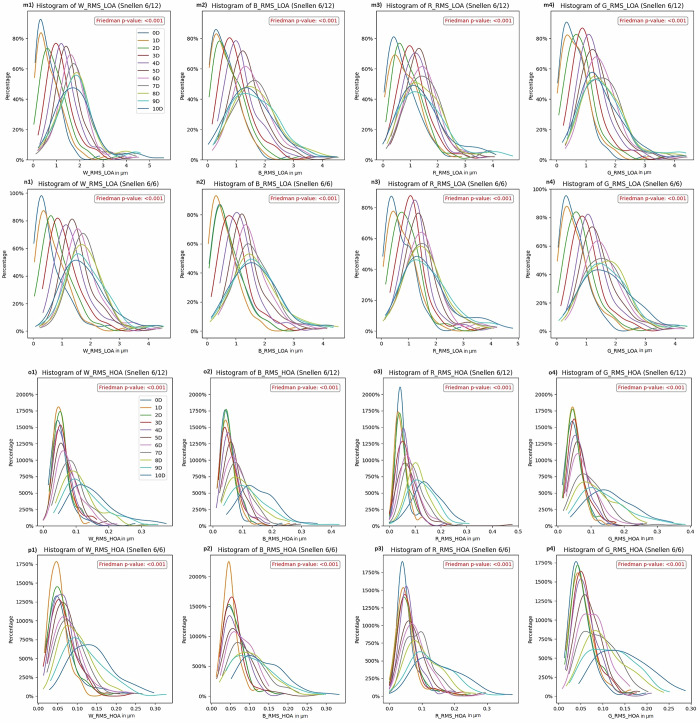
(Continued)

Amongst the analysed Zernike coefficients, the defocus coefficient, *C*(2,0), demonstrated the largest variation in response to increasing accommodative demand, for both 6/6 and 6/12 target sizes (Fig. 2, d for the TG; Fig. 3c, d for G1). In contrast, the coefficients associated with astigmatism, oblique *C*(2,−2) and vertical *C*(2,2), showed smaller changes. Nonetheless, both coefficients revealed statistically significant differences at specific accommodative levels. In the younger group (G1), the oblique astigmatism coefficient (Fig. 3a, b) reached significance (*p* < 0.0009) in four out of the eight measurements, while vertical astigmatism (Fig. 3e, f) showed significance in two. When considering the TG, statistically significant differences in *C*(2,−2) (*p* < 0.003) were observed across all filters except for the white one, and *C*(2,2) also varied significantly across conditions, although to a lesser extent (Fig. 2a, b, e, f).

Regarding HOA, vertical coma *C*(3,−1) did not exhibit significant variations in response to increased accommodative demand in G1 (Fig. 3g, h), and only showed significant changes in four out of the eight accommodative steps in the TG analysis (Fig. 2g, h). In contrast, horizontal coma *C*(3,1) showed consistent and significant differences under all tested conditions for both the G1 and the TG (Figs. 2i, j and 3i, j), regardless of the filter applied or the stimulus size. In G1, a distinct trend was observed for the white and blue filters: horizontal coma increased between 1 and 4 D and then decreased at higher accommodative demands. Conversely, for the red and green filters, the largest values were recorded at the lowest accommodative levels, followed by a progressive decline.

Analysis of the spherical aberration coefficient, *C*(4,0), revealed significant variations associated with increasing accommodative demand in both G1 and the TG (Figs. 2k, l and 3k, l). In G1, significant differences (*p* < 0.0009) were observed across all filters and target sizes. A consistent pattern emerged: a progressive shift toward more negative values occurred between 0 and 5 D, followed by a return toward less negative or even positive values at higher demands (6–10 D). In contrast, in the TG, which was only evaluated up to 5 D, significant changes (*p* < 0.003) were found in three of the eight conditions. While the general trend mirrored that of G1, i.e., an initial shift toward negative spherical aberration, one exception was noted with the blue filter and 6/12 target; the final accommodation steps showed a reversal toward positive values.

**Fig. 4:**
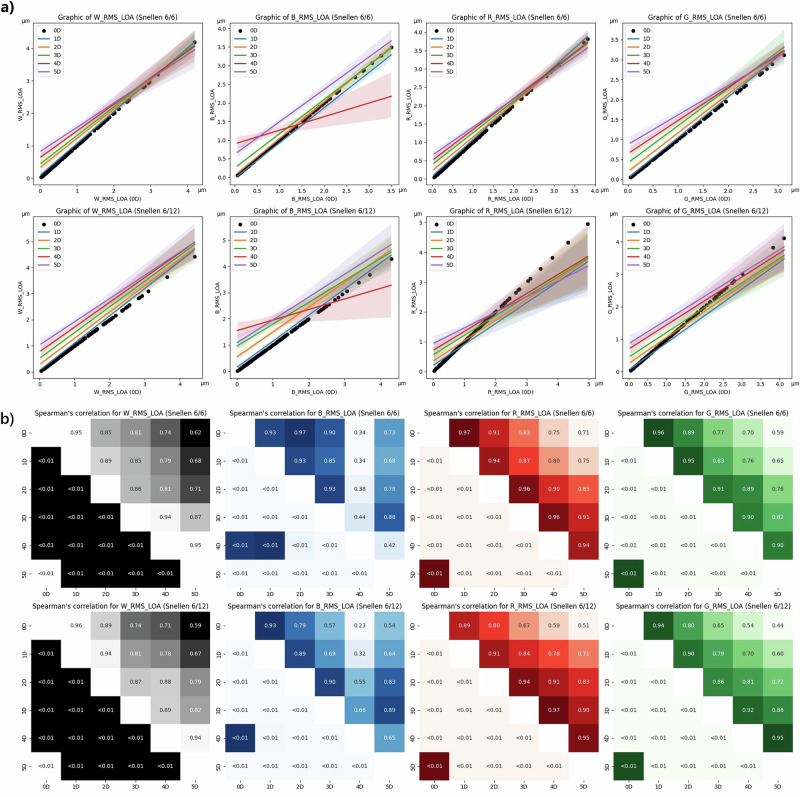
Low-order aberration (LOA) root mean square (RMS) changes and correlations across accommodative demand for the total group (TG). **a** Linear regressions of RMS values in micrometres (µm) for LOAs for each accommodative step in dioptres (D), from 0 to 5 D, compared to the unaccommodated eye, according to chromatic filter (white, blue, red and green) for the TG. **b** Spearman correlation matrices between accommodative demands for LOA RMS values, with correlation coefficients shown in the upper diagonal and corresponding *p* values (statistically significant when *p* < 0.05) in the lower diagonal, organised by chromatic filter (white, blue, red and green). In both subfigures, the top row corresponds to the small optotype stimulus (Snellen E 6/6) and the bottom row to the large optotype stimulus (Snellen E 6/12).

Finally, to evaluate the overall magnitude of accommodation-induced optical aberrations, the progression of root mean square (RMS) values for LOA and HOA was analysed across all target sizes and filters. In both G1 and TG, statistically significant differences were observed between accommodative levels (*p* < 0.0009 for G1; *p* < 0.003 for TG). In G1, LOA RMS values exhibited a consistent percentage decrease and a shift toward more positive values up to approximately 6 D, followed by a slight return toward less positive values at higher demands (7–10 D) (Fig. 3m, n). Similarly, HOA RMS values tended to increase from 1 to 4 D relative to baseline (0 D), then decreased beyond 5 D in all cases (Fig. 3o, p). This pattern was observed across all filters except for the red filter, where the HOA RMS remained more stable. In the TG, which was only analysed up to 5 D, the same general trends were observed, although less pronounced due to the limited range of accommodation. Statistically significant differences (*p* < 0.003) were found across all conditions studied, regardless of the filter or target size used. As shown in Fig. 4, both RMS values showed a percentage decrease and a shift toward more positive values, with this trend being more pronounced for LOA RMS.

### Relationships Between Accommodation States: Correlations and Regressions

**Fig. 5:**
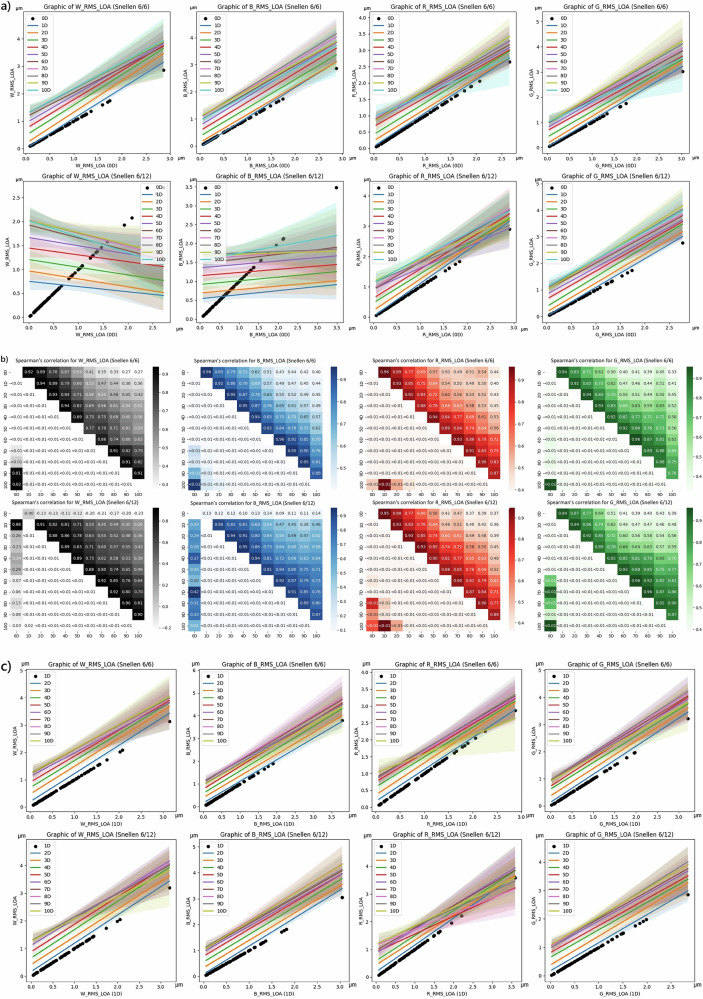
Low-order aberration (LOA) root mean square (RMS) modulation and correlations across extended accommodative demand in the young group (G1). **a** Linear regressions of RMS values in micrometres (µm) for LOAs at each accommodative step in dioptres (D), from 0 to 10 D, compared with the unaccommodated state, separated by chromatic filter (white, blue, red and green) for the G1. **b** Spearman correlation matrices of LOA RMS values across accommodative demands, with correlation coefficients shown in the upper diagonals and *p* values (statistically significant at *p* < 0.05) in the lower diagonals, for each chromatic filter. **c** Linear regressions of LOA RMS values at each accommodative step using 1 D as the reference, by chromatic filter (white, blue, red and green). In all panels, the top row corresponds to the small optotype stimulus (Snellen E 6/6) and the bottom row to the large optotype stimulus (Snellen E 6/12).

Figures 4a, b and 5a, b illustrate the optical response of LOAs as a function of accommodative demand, analysed through linear regression and correlation matrices. In both the TG and the younger group (G1), the linear regression models comparing RMS LOA values at each accommodative level with the baseline (0 D) revealed a progressive reduction in the slope of the regression lines with increasing accommodative effort. This trend was especially pronounced with the white and blue filters for the larger 6/12 target, where the slopes became markedly flatter. In the case of the white filter, the slope even became negative in G1, suggesting a potential overestimation of accommodative effort during the initial responses. Correlation matrices confirmed this finding, showing reduced or absent correlation in these conditions, particularly for G1 with the white and blue filters and the 6/12 target, reinforcing the hypothesis of early-stage accommodative inefficiency or variability. Interestingly, when considering the spectral characteristics and luminance levels of each filter, particularly the white and blue filters, which closely resemble daylight and are perceived as brighter, an initial overaccommodation may have occurred. This would explain the flatter or even negative regression slopes in the early accommodative steps, followed by a recalibration as demand increased. This hypothesis was further supported by regression analyses comparing each accommodative level with the 1 D baseline (Fig. 5c), where similar response trends were observed across all filters and target sizes.

Figures 4b and 5b complement these results by presenting Spearman correlation matrices comparing RMS LOA values across all accommodative conditions. A strong correlation was found between responses to consecutive accommodative demands, progressively weakening as the distance between stimuli increased. This indicates that optical response similarity is highest when accommodative stimuli are close in magnitude. This trend was more pronounced for LOA values, while HOA correlations followed a similar but less marked pattern, likely due to increased variability as the Zernike order increased (Supplementary Material Figs. S1 and S2). Comparable behaviour was also observed for the Total RMS values, which closely mirrored the LOA RMS, given that HOA RMS contributed only minimal modifications (Supplementary Material Figs. S3 and S4).

### Analysis of the Actual vs. Theoretical Accommodation Curve

**Fig. 6:**
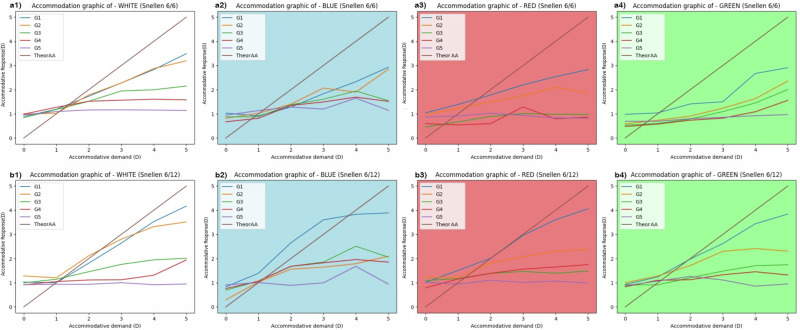
Measured accommodative response as a function of accommodative demand from 0 to 5 D under different chromatic filters: white, blue, red and green. The graphs represent five age-based groups: G1 (20–29 years), G2 (30–39 years), G3 (40–49 years), G4 (50–59 years) and G5 (60–75 years), compared against the theoretical accommodative amplitude (TheorAA). The top row corresponds to measurements using the small optotype (Snellen E 6/6) and the bottom row to the large optotype (Snellen E 6/12).

**Fig. 7:**
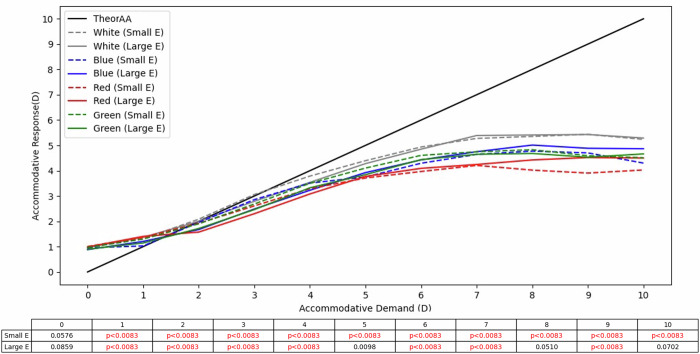
Measured accommodative response curves (in dioptres) relative to the theoretically induced accommodative demands for the four chromatic filters (white, blue, red and green) and two optotype sizes (large: Snellen E 6/12; small: Snellen E 6/6) from 0 to 10 D, shown as mean responses across participants in the young group (G1).The black line represents the theoretical accommodative demand (TheorAA). Solid coloured lines depict responses for each chromatic filter with the 6/12 optotype, while dashed lines represent responses with the 6/6 optotype. The bottom table displays the *p* values for statistical comparisons between the four filters using large optotypes (6/12) and between the four filters using small optotypes (6/6), at each level of accommodative demand. Red values indicate statistically significant differences (Friedman test with Bonferroni correction, 0.05/6: *p* < 0.008).

Based on the values of the defocus coefficient *C*(2,0) and spherical aberration *C*(4,0) obtained at each accommodative step, the actual accommodative response curves obtained for each stimulus condition (white, blue, red and green filters) across two target sizes (6/12 and 6/6) were derived and compared to the theoretical accommodative demand (Figs. 6 and 7), by evaluating the *M* value (Eq. 1) at each level of stimulation, with *r* = pupil radius.

1 $$M=\frac{C\left(2,0\right)* 4\sqrt{3}-C\left(4,0\right)* 12\sqrt{5}}{{r}^{2}}$$

Data were collected from 164 subjects divided into five age groups, with accommodative demands ranging from 0 to 5 D (Fig. 6). In this case, the white filter consistently induced the greatest accommodative response along the entire curve. At the final demand level of 5 D, the accommodative response with the white filter reached the following values (for the 6/12 target, Fig. 6b1): G1: 4.69 D; G2: 3.52 D; G3: 2.01 D; G4: 1.94 D and G5: 0.95 D. For the 6/6 target (Fig. 6a1), the values were: G1: 3.38 D; G2: 3.19 D; G3: 2.15 D; G4: 1.58 D and G5: 1.14 D. Conversely, the red filter generated the lowest responses, indicating under-accommodation across all groups and target sizes. For the 6/12 target (Fig. 6b3), responses were: G1: 4.43 D; G2: 2.38 D; G3: 1.48 D; G4: 1.75 D and G5: 0.93 D, and for the 6/6 target (Fig. 6a3): G1: 2.98 D; G2: 1.30 D; G3: 0.98 D; G4: 0.86 D and G5: 0.82 D. Responses under the blue and green filters fell between these two extremes (Fig. 6a2, b2, a4, b4).

Younger groups (G1 and G2) showed a significantly greater accommodative response, more closely approximating the theoretical values under all conditions, whereas older groups (G4 and G5) exhibited a nearly flat response. Additionally, the larger stimulus (6/12, Fig. 6b) elicited greater accommodation across all age groups. In addition, within the 6/12 condition, the blue filter generated a distinct response pattern in G1 (Fig. 6b2). Beyond the general overaccommodation noted previously, this group showed a localised inflexion in the response curve, indicating greater sensitivity to short-wavelength stimulation compared with the other filters. This deviation appeared exclusively in G1, consistent with the higher accommodative reactivity and wider dynamic range typically found in younger subjects.

Additionally, more detailed measurements in G1 (*n* = 82) extended the accommodative demand up to 10 D (Fig. 7). Across all groups and conditions, subjects showed an over-accommodated baseline at 0 D, with an approximate accommodative response of 1 D. In G1, a clear decrease in accommodative response was observed from 5 to 6 D onwards, regardless of stimulus colour or size, indicating physiological limitations in sustaining higher accommodation (Fig. 7). This decline beyond 5 D was not evaluated in older groups due to their limited accommodative capacity. The maximum accommodative responses obtained with aberrometry were substantially lower than the subjective AA reported in Table 1. This discrepancy is expected because the subjective push-up test was performed briefly, under photopic and open-field conditions that tend to overestimate AA, whereas the aberrometry measurements required sustained accommodation for 22 s under scotopic illumination and closed-field viewing, conditions that induced fatigue and reduced accommodative performance.

In G1, a greater accommodative response was observed with larger visual stimuli (6/12) compared to smaller ones (6/6), across all chromatic filter conditions. This trend was particularly marked at higher levels of accommodative demand (>7 D). The four chromatic filters were compared at each level of accommodative demand and for each target size separately. Statistically significant differences between filters (*p* < 0.008) were found for most accommodative levels (see p-value table in Fig. 7). Exceptions occurred at baseline (*p* = 0.06 and 0.09 for the 6/6 and 6/12 targets, respectively) and at higher accommodative demands (8–10 D) for the larger 6/12 target, where differences between filters diminished (*p* = 0.05 and 0.07 at 8 and 10 D, respectively). Although statistical comparisons between age subgroups were not included in Fig. 7 for the 0–5 D accommodative range, the same trend was observed across all age groups, with consistent patterns in accommodative behaviour under the different chromatic conditions.

## Discussion

The present findings indicate that the accommodative response and pupillary constriction are modulated by both chromatic content and target size, with younger participants (G1) showing a broader dynamic range and greater responsiveness. Variations in Zernike coefficients revealed that defocus (*C*(2,0)) and spherical aberration (*C*(4,0)) were particularly sensitive to changes in stimulus characteristics. Responses were evaluated across participants aged 20–75 years, with demands up to 5 D for all and up to 10 D for G1, highlighting how age and optical conditions interact to shape accommodative performance. These results extend previous aberrometry studies [31, 35, 45–50] by examining responses under varied lighting conditions, which have not been explored sufficiently to date, and under higher accommodative demands than typically reported.

Regarding pupil size, pupil diameter decreased with accommodation and increased with longer wavelengths. Additionally, smaller visual stimuli, by increasing the effective retinal illuminance, tended to induce greater pupillary miosis, a response that enhances depth of focus and VA [3, 51]; findings that are consistent with the results obtained in this study. Across all tested conditions, a progressive decrease in pupil diameter was observed as accommodative demand increased. The average 2 mm pupillary constriction during accommodation indicates a strong accommodative response, particularly in younger subjects with greater ciliary muscle and pupillary contractility. The room was kept under scotopic conditions, with the stimulus (through its spectral filter) as the only light source, which likely explains the relatively large pupil sizes even under high accommodative demands. This trend persisted using the 5th percentile criterion (Tables 2 and 3), with the smallest pupils in G1 (*n* = 82) under 10 D of accommodative demand using the white filter (3.05 mm for the 6/12 stimulus and 3.03 mm for the 6/6 stimulus) and a minimum of 3.65 mm at 5 D with the green filter (6/6 target), consistent with previous studies [32] and theoretical models describing pupillary behaviour under accommodative stimulation [52].

Concerning the Zernike coefficients, the literature indicates that the aberration terms most consistently reported to vary with accommodation are primarily vertical astigmatism *C*(2,2), defocus *C*(2,0), oblique astigmatism *C*(2,−2), vertical coma *C*(3,−1), horizontal coma *C*(3,1) and spherical aberration *C*(4,0) [1, 22, 23, 25, 29, 43, 44]. Nevertheless, such variations may depend on factors including the number of participants per age group and experimental condition (sample size), participant age, type of stimulus, the method used to induce accommodation and the specific aberrometer being employed. In the present study, the defocus coefficient *C*(2,0) exhibited the largest variations, aligning with previous reports up to 5 D [1, 23, 53], increasing with accommodation. At higher demands (6–10 D) in younger subjects, the increase in defocus plateaued or even decreased, likely due to concurrent changes in spherical aberration, *C*(4,0) and the biomechanical limits of the crystalline lens in continuing to increase its power under high accommodative stress [24].

Vertical coma *C*(3,−1) remained stable throughout accommodation, consistent with previous studies [22, 24, 36, 49], possibly due to relaxation of the lens zonules during accommodation, which may reduce lens decentration and stabilise vertical coma [24]. In contrast, horizontal coma *C*(3,1) exhibited a progressive increase with accommodation, indicating greater sensitivity to structural changes in the lens. However, findings are mixed, with some studies supporting stability and others observing significant variation [53], while coma coefficients generally show no systematic pattern or relative stability during accommodation [24].

The spherical aberration coefficient *C*(4,0) exhibited a pattern consistent with previous literature, becoming increasingly negative as accommodative demand rose to 5 D [1, 53]. This behaviour, related to the sign reversal of spherical aberration during accommodation and its impact on the relative position of paraxial and marginal foci, has been described in prior studies [54]. Above 5 D, *C*(4,0) shifted progressively towards more positive values [55], likely due to the crystalline lens reaching its deformation limit while ciliary contraction and pupillary constriction continue, producing a compensatory optical effect that increases spherical aberration [24].

LOA progressively modulated with increasing accommodation, with younger participants showing reduced slope variability, particularly under the blue filter with the 6/12 stimulus (Figs. 4a and 5a). Stronger correlations between adjacent demands indicated more consistent LOA responses during gradual changes (Figs. 4b and 5b), while *C*(2,0) and *C*(4,0) primarily drove differences across filters and stimulus sizes (Figs. 2m–p and 3m–p). In G1, the 6/12 optotype under the white and blue filters exhibited initial overaccommodation, reflected by flat or negative slopes and weak correlations, likely due to higher luminance and spectral content resembling daylight [15]. This effect was specific to larger targets, whereas smaller stimuli required more precise responses [51]. Comparisons with the 1 D accommodative state and the full sample confirmed the same trend from 0 D for all filters, reinforcing that overaccommodation is particularly prominent in younger individuals [56].

Accommodative responses across age groups consistently fell below the theoretical demand, indicating a progressive accommodative lag, which was less pronounced under the white filter, consistent with more efficient accommodation under broad-spectrum light [3, 57]. Previous studies also showed greater AA under blue light, followed by green and then red, due to the increased depth of focus at shorter wavelengths [14]. Under the red condition, the low luminance (5 cd/m²) likely caused larger pupils and reduced accommodative responses, which explains why the accommodative response curve under the red filter is flatter than the others, reflecting lower accommodative efficiency.

Regarding stimulus size, an inversion in accommodative efficiency was observed depending on the level of demand. In the early stages of the accommodation curve (up to 4–5 D), the smaller optotype (6/6) elicited a more effective response. This is likely because its higher spatial frequency requires more precise focusing, thereby stimulating a more active accommodative control [51]. However, as accommodative demand increased, this trend reversed: the larger optotype (6/12) provides better visual discrimination under defocus due to its lower spatial frequency, which facilitates the maintenance of accommodative effort. These findings align with previous studies [3, 51], showing that larger targets elicit stronger accommodative responses and greater pupillary constriction at high demands, whereas smaller targets enhance focusing precision at low demands, clarifying that the effects of optotype size are complementary rather than contradictory. Furthermore, recent research has reinforced the influence of wavelength and optical design on accommodative behaviour. Aissati et al. [58]. demonstrated that novel optical designs modulate both the accommodative response and visual performance under varying optical conditions, while Rucker and Kruger [59] integrated the effects of colour (wavelength) and visual stimulus properties, highlighting the role of short-wavelength-sensitive cones and chromatic aberration in accommodation. Together with the findings of Fernández-Alonso et al. [3], these studies support the interaction between light wavelength, optical characteristics and target size in shaping accommodative mechanisms. Initially, the accommodative responses are similar for both optotypes; yet, as the demand rises, the differences become more pronounced. This suggests that at higher accommodation levels, stimulus detectability outweighs focusing precision, indicating a shift in the visual system’s prioritisation from acuity to sustained engagement under increased optical stress. These results have potential implications for both visual ergonomics and clinical practice. In occupational and digital environments, the use of shorter-wavelength or broadband illumination may support more accurate accommodative responses and improved visual comfort. In addition, accommodation training strategies could be refined by incorporating both stimulus size and chromatic cues, thereby enhancing the effectiveness of therapeutic interventions, particularly in early presbyopia.

An initial overaccommodation was noted across all filters and stimulus sizes, reflecting instrumental accommodation [60]. The influence of the chromatic content was evident: broad-spectrum illuminants (white and blue) appeared to elicit stronger and more efficient accommodative activation, while monochromatic filters, particularly red, may limit accommodative performance by reducing LCA, a known cue for accommodation [1, 14].

The present results align with those of Fernandez-Alonso et al. [3], who controlled luminance strictly across illuminants. Both studies found that accommodation remains effective under narrowband light and that chromatic aberration compensation occurs mainly at near distances. In the current findings, white and blue light induced greater pupil constriction and stronger accommodative responses than red and green, particularly with larger stimuli. Although luminance was not equalised to preserve spectral purity and ecological validity, the findings suggest that the observed effects were driven primarily by the spectral composition of light rather than luminance differences.

As expected, age affected the accommodative response significantly: younger participants (G1 and G2) displayed steeper and more dynamic curves, aligning with theoretical demand, particularly under white and blue lighting, while older groups (G4 and G5) exhibited flatter, less variable responses across lighting conditions or optotype sizes, consistent with the presbyopic decline resulting from lens rigidity and reduced ciliary muscle function [26, 61].

This study has limitations, including the use of non-standard pupil diameters (3.00 and 3.65 mm), which complicated comparisons with previous studies. A 3.00 mm pupil diameter was used in the youngest group for responses up to 10 D, while a 3.65 mm pupil diameter was applied across all groups for demands up to 5 D. This methodological choice helped preserve data quality at higher levels of accommodation and allowed comparisons across conditions; however, it necessarily reduced the physiological variability of pupil-linked aberrations. As HOAs are particularly sensitive to pupil size, this limitation should be considered when interpreting the results. In addition, accommodative responses may be slightly overestimated, since Seidel defocus is known to bias accommodation measurements [62].

The sample showed a relatively balanced sex distribution (78 males and 86 females), with no reported sex-related differences in accommodation [45]. Most participants were myopic (136 subjects), with fewer emmetropes (15 subjects) and hyperopes (13 subjects), which could have influenced the results since myopes tend to exhibit lower AAs [63–65].

A further limitation is that only whole-eye aberrations were analysed, without differentiating corneal and lenticular contributions, limiting the interpretation of certain changes in Zernike coefficients. The fixed interval of 2 s between accommodative steps may have also added variability, as individual accommodation times differ. Although similar intervals have been used before [51, 53], different stimuli may require adjusted timing, suggesting future studies should adopt a more personalised approach.

Finally, although chromatic filters with different spectral properties were used, luminance levels were not equalised across them. This discrepancy may have affected both stimulus perception and the magnitude of the accommodative response, particularly under high-luminance conditions, which limits direct comparisons across chromatic conditions, especially when overlapping regions of spectral irradiance occur in different parts of the spectrum.

In conclusion, under the specific conditions of this study, accommodation efficiency was influenced by light colour and stimulus size. White and blue illumination induced greater pupil constriction and stronger accommodative responses than red and green light. Aberrometric changes, particularly in defocus and spherical aberration, reflected these dynamics. Smaller stimuli enhanced accommodation at low demands, while larger stimuli were more effective at high demands. A progressive accommodative lag appeared for stimuli >5 D, smallest under white and largest under red light. Young subjects showed initial overaccommodation under white and blue light with larger stimuli. Overall, these findings suggest that spectral and spatial visual conditions can modulate the efficiency and quality of the accommodative response.

## Supplementary Information

Supplementary Information

## Data Availability

The data that support the findings of this study are available from the corresponding author upon reasonable request.
