# Supplementary material for: Influence of Light Wavelength and Optotype Size on Accommodative Response and Aberrometric Changes Across the Adult Lifespan
Source: Ophthalmic Physiol Opt. 2026 Mar 9;46(2):211–29. doi: 10.1007/s44402-026-00036-0 (PMC13369024; doi:10.1007/s44402-026-00036-0)
Supplement: Supplementary file 1 — Supplementary Information [file 44402_2026_36_MOESM1_ESM.docx]

**SUPPLEMENTARY MATERIAL**


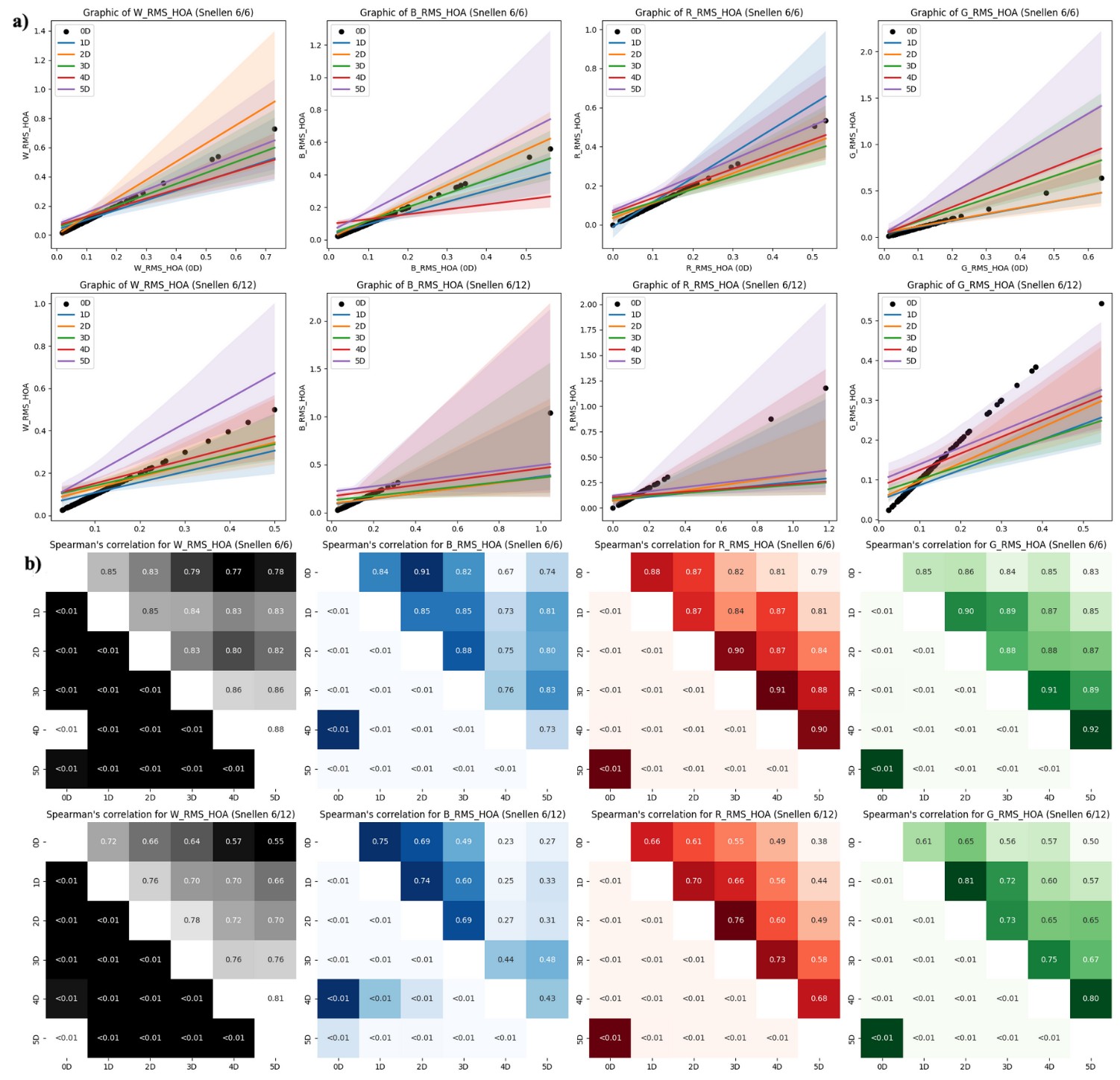


**Supplementary Fig. S1.**

**High-order aberration (HOA) Root Mean Square (RMS) changes with accommodative demand in the total group (TG).** (a) Linear regressions of RMS values in micrometres (µm) for HOAs for each accommodative step in dioptres (D), from 0 to 5 D, compared to the unaccommodated eye, according to chromatic filter (white, blue, red, and green) for the TG. (b) Spearman correlation matrices between accommodative demands for HOA RMS values, with correlation coefficients shown in the upper diagonal and corresponding p-values (statistically significant when *p* < 0.05) in the lower diagonal, organized by chromatic filter (white, blue, red, and green). In both subfigures, the top row corresponds to the small optotype stimulus (Snellen E 6/6) and the bottom row to the large optotype stimulus (Snellen E 6/12).


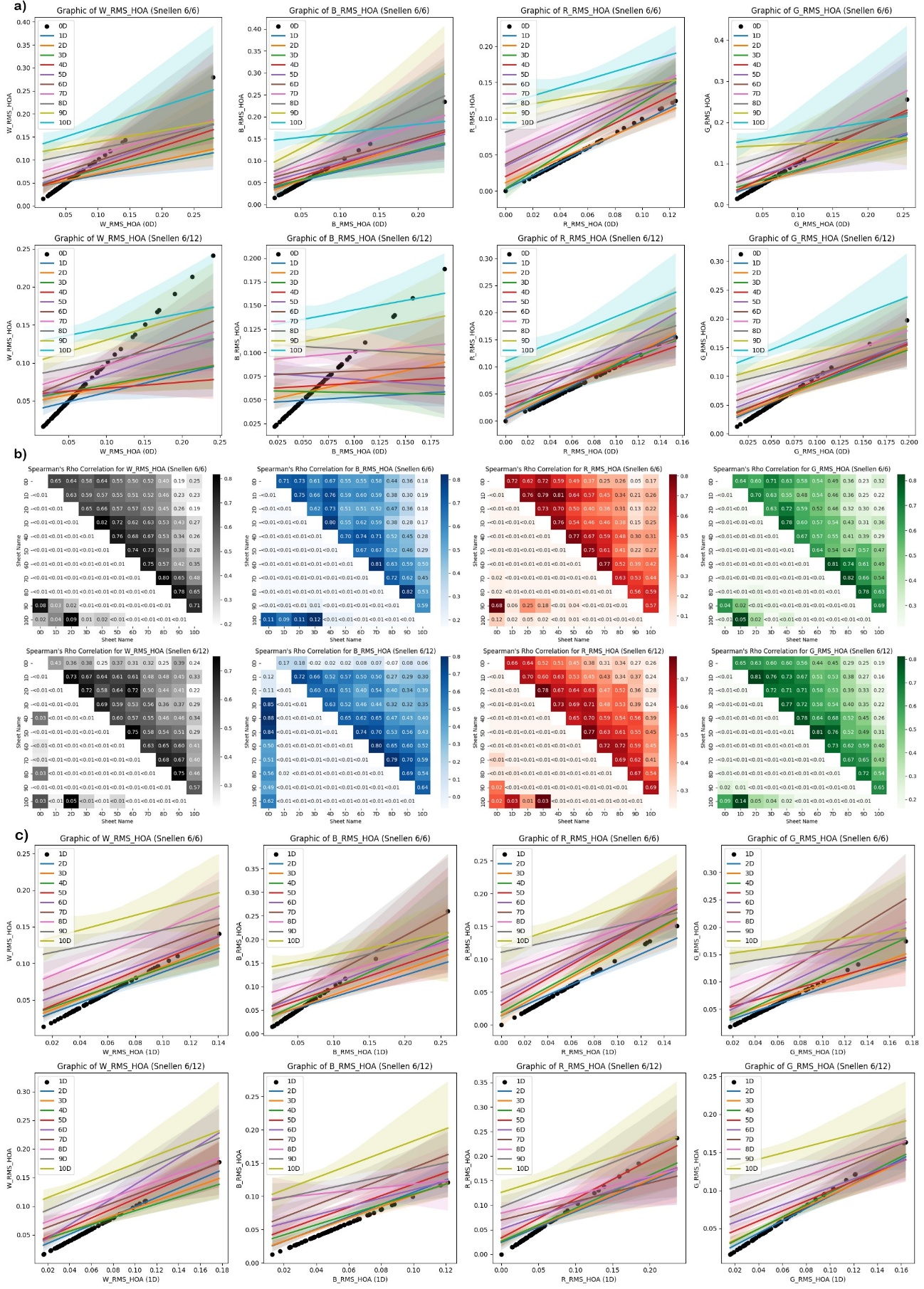


**Supplementary Fig. S2.**

**High-order aberration (HOA) Root Mean Square (RMS) behavior across extended accommodative demands in the young group (G1).** a) Linear regressions of RMS values in micrometres (µm) for HOAs at each accommodative step in dioptres (D), from 0 to 10 D, compared to the unaccommodated state, separated by chromatic filter (white, blue, red, and green) for the G1. b) Spearman correlation matrices of HOA RMS values across accommodative demands, with correlation coefficients shown in the upper diagonals and p-values (statistically significant at *p* < 0.05) in the lower diagonals, for each chromatic filter. c) Linear regressions of HOA RMS values at each accommodative step using 1 D as the reference, by chromatic filter (white, blue, red, and green). In all panels, the top row corresponds to the small optotype stimulus (Snellen E 6/6), and the bottom row to the large optotype stimulus (Snellen E 6/12).


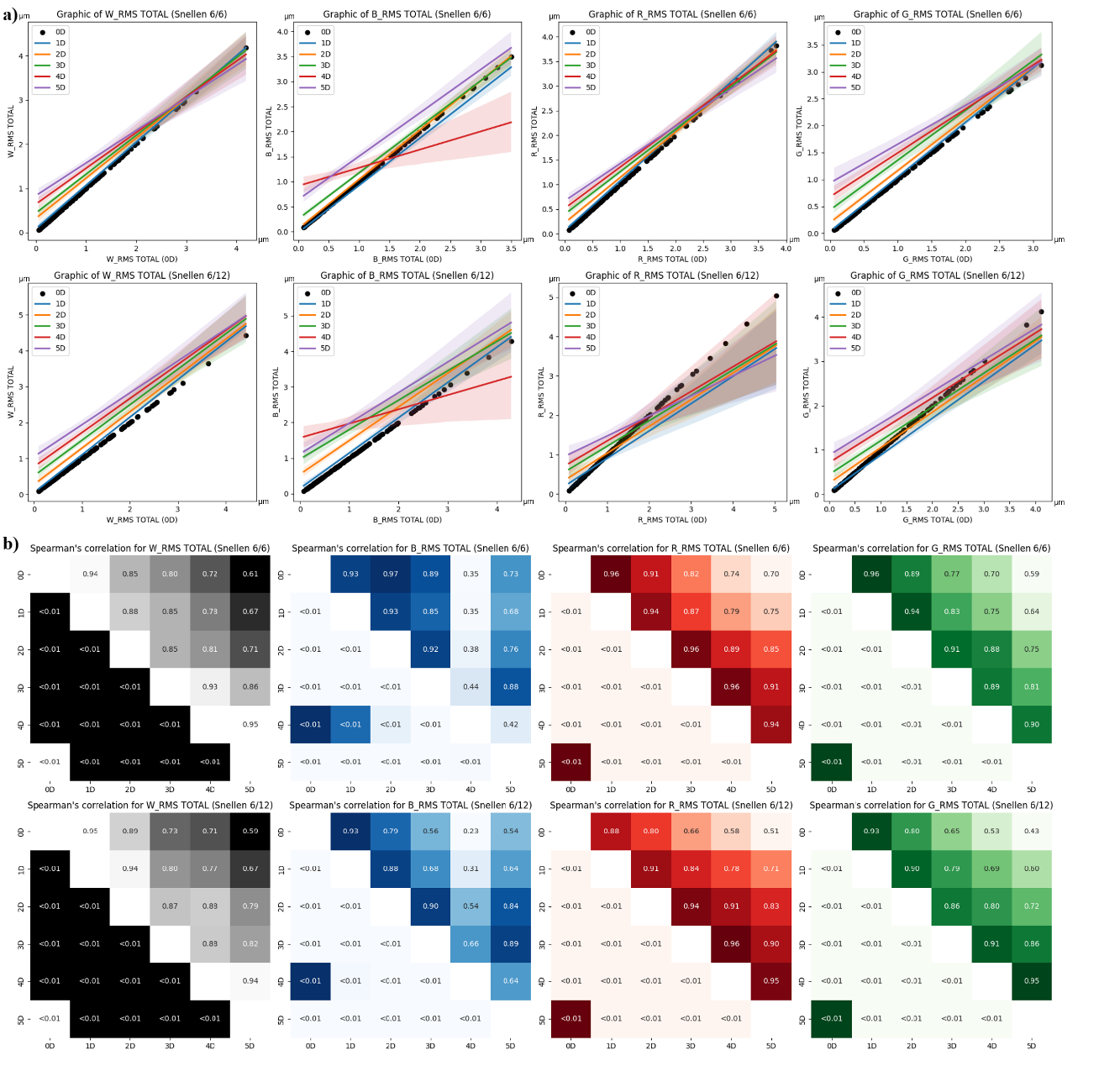


**Supplementary Fig. S3.**

**Total aberration (LOAs and HOAs) Root Mean Square (RMS) changes with accommodative demand in the total group (TG).** (a) Linear regressions of RMS values in micrometres (µm) for total aberrations (LOAs and HOAs) for each accommodative step in dioptres (D), from 0 to 5 D, compared to the unaccommodated eye, according to chromatic filter (white, blue, red, and green) for the TG. (b) Spearman correlation matrices between accommodative demands for Total RMS values, with correlation coefficients shown in the upper diagonal and corresponding p-values (statistically significant when *p* < 0.05) in the lower diagonal, organized by chromatic filter (white, blue, red, and green). In both subfigures, the top row corresponds to the small optotype stimulus (Snellen E 6/6) and the bottom row to the large optotype stimulus (Snellen E 6/12).


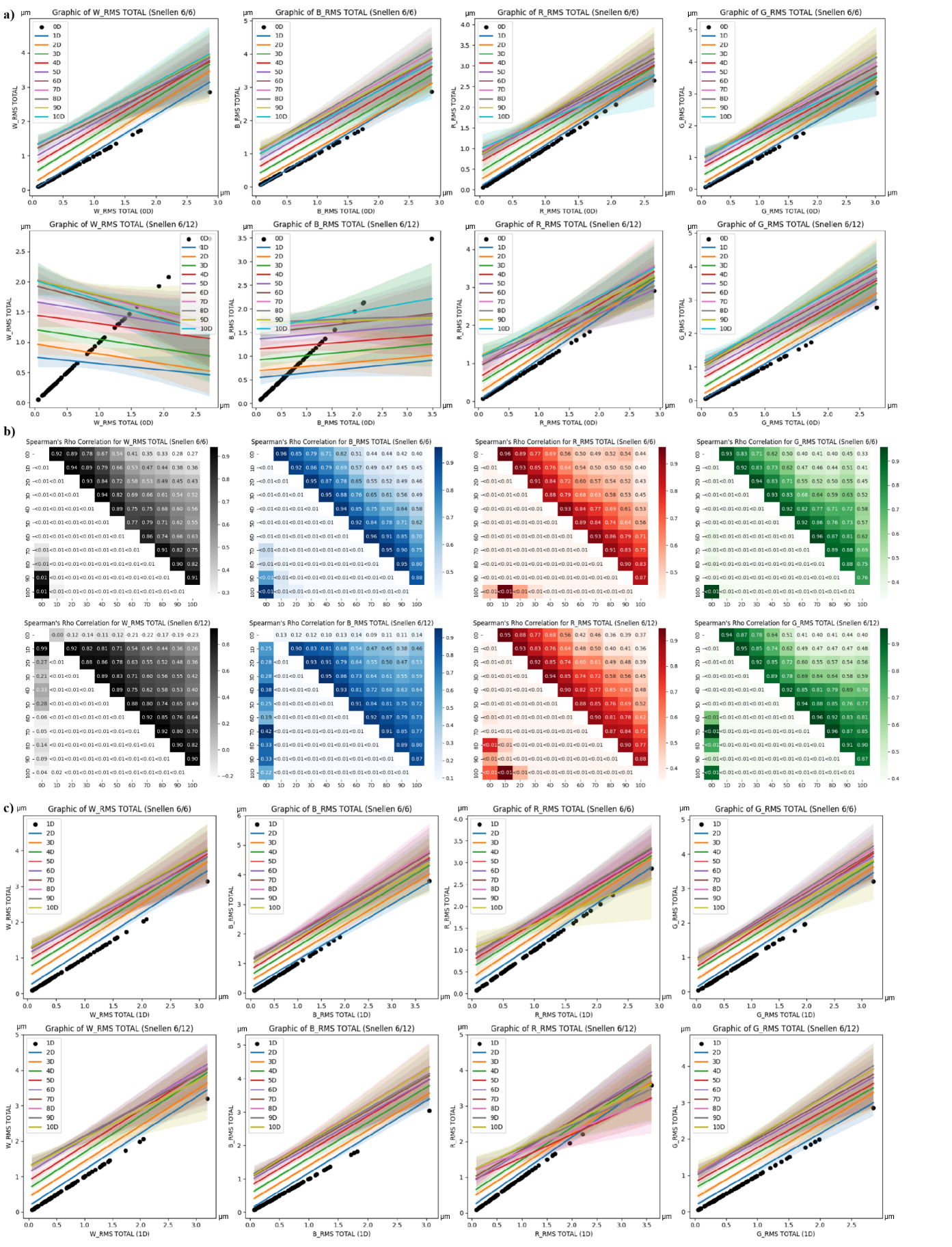


**Supplementary Fig. S4.**

**Total aberration (LOAs and HOAs) Root Mean Square (RMS) behavior across extended accommodative demands in the young group (G1)**. a) Linear regressions of RMS values in micrometres (µm) for total aberrations (LOAs and HOAs) at each accommodative step in dioptres (D), from 0 to 10 D, compared to the unaccommodated state, separated by chromatic filter (white, blue, red, and green) for the G1. b) Spearman correlation matrices of Total RMS values across accommodative demands, with correlation coefficients shown in the upper diagonals and p-values (statistically significant at *p* < 0.05) in the lower diagonals, for each chromatic filter. c) Linear regressions of Total RMS values at each accommodative step using 1 D as the reference, by chromatic filter (white, blue, red, and green). In all panels, the top row corresponds to the small optotype stimulus (Snellen E 6/6), and the bottom row to the large optotype stimulus (Snellen E 6/12).
